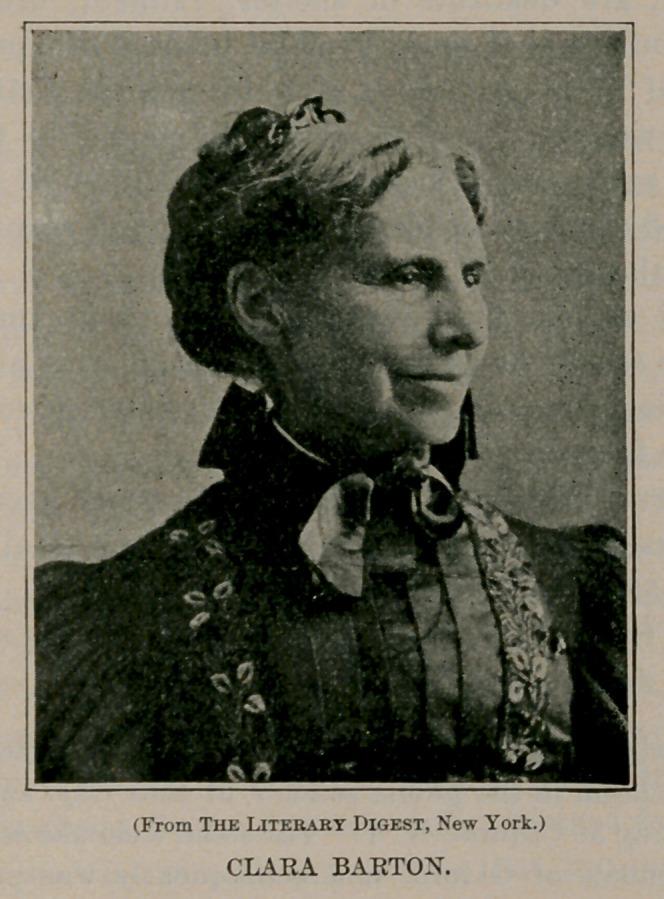# Topics of the Month

**Published:** 1896-12

**Authors:** 


### TOPICS OF THE MONTH.

The Dental Department of the University of Buffalo opened its new building, situated on the university grounds facing Goodrich street, on Tuesday evening, October 20, 1896. The ceremonies included a prayer by the Rev. Thomas R. Slicer, the formal transfer of the property by the contractors, Henry Schaffers' Sons, an address by Dr. W. C. Barrett, dean of the faculty, an address by Hon. James O. Putnam, chancellor of the University, and remarks of congratulation by Dr. M. D. Mann, dean of the Medical Department.

The feature of the evening was an address by Mr. Melvil Dewey, state librarian and secretary of the regents of the Uni versity of the State of New York. Mr. Dewey was very cordially received and spoke in part as follows:

There are four reasons why I have chosen as the topic of my remarks this evening, the subject, Buffalo as an Educational Center.

The first of these reasons is its geographical situation, which fits it to be the center of everything. Even the most casual observer can see that its position is one of especial advantage, and that it occupies a remarkable location on this planet.

The second reason is that Buffalo is the second city in the State of New York, and that this state has the best educational facilities of the United States, and that the time is coming when no spot on the globe will be better educated than the Empire State. The time is not far distant when we shall draw students from other countries, as our students now go abroad.

The third reason for my choice of a topic is that Buffalo has done so much, and my fourth reason is that it has done so little.

My principal theme will be the second reason which I have given, and I propose to tell some of the reasons why New York is the best State in the Union from the viewpoint of education, and something of the work that is being done by the University of the State of New York, under the direction of its regents.

Mr. Dewey then went into an exhaustive history of the foundation and organisation of the university of the state, and dwelt on the excellence of the laws governing it and the work that is being done now by it along the lines of higher education. He dwelt particularly on the working value of the educational institutions that are not generally classed as such—the libraries, the study clubs, the museums and the university-extension lectures under the direction of the regents of the university. Returning to the third and fourth reasons he had given for the choice of his subject, he said:

The reason for my third purpose is that the famė of the Buffalo medical school is enviable, and competent physicians who have examined it speak with warm approval of the work that is being done here. The same may be said of the other institutions of learning in this city. You will wonder why, then, I stated my fourth reason to be that Buffalo has done so little. I will tell you. Buffalo has done little for the cause of education in that she has not yet a free public library. The time is upon us when a citizen of a village or a town will be just as mortified to confess that his home contains no free library as he would be to admit that there were no free public schools. It would cost too much, is the plea. The number of cents each taxpayer would have to pay to make your magnificent library free to the public, absolutely free, is so small that it is not worthy of consideration. Then, there is one more cause for grief on Buffalo's behalf in that she has not yet endowed her University. It ought to be known that it pays commercially to maintain educational institutions, and I hope that Buffalo is merely waiting for the time to be ripe and not sitting in heedless lethargy and paying no attention to this great and vital subject.

At the conclusion of Mr. Dewey's address a vote of thanks to the speaker was adopted unanimously, and the visitors were invited to inspect the new building and its fittings.

---

THAT Buffalo needs a free public library goes without saying. The only wonder is that it has not been settled upon long before this. Our citizens ought to be very thankful to Mr. Dewey for calling attention to this important factor in education and to the necessity of its speedy establishment. The additional cost of converting the Buffalo library into one that would be absolutely free to the public is so trifling in comparison with the results that would follow that our city fathers need not hesitate to incur it. "It ought to be known that it pays commercially to maintain educational institutions," says Mr. Dewey. This was a most happy way to present the matter, since it is about the only avenue of approach to the average mind in the city legislature. Let us have a free city library, by all means, Mr. Common Councilman, and thus testify that we intend to keep intellectual light abreast of electric illumination. We are quite content to have Niagara furnish the power for the latter, but let Buffalo herself furnish the motive force for the other.

---

THE moment the Buffalo library is made free it will receive contributions of books, as well as money, that will constantly increase its efficiency and value. In this connection we are pleased to note that Dr. De Laskie Miller, of Chicago, according to the *Medical Recorder*, has lately given his entire collection of books to the Newberry library. Professor Miller, a native, we believe, of Niagara county, has succeeded in collecting one of the most valuable private medical libraries in Chicago, composed principally of standard works, ancient and modern, and it is such a library as well might be the envy of any physician. How long will it be before we shall hear of similar contributions to the Buffalo free library?

---

The State Board of Health has formulated new blanks for use in the bureau of vital statistics. These appear to be an improvement on the old form, being simpler and yet containing provisions for more information. They will result in great saving to the health department in this city, inasmuch as the blanks are to be bound in book form as they are filed. This will obviate the necessity of manufacturing expensive record books in which to transcribe them.

---

The new law regarding the employment of children in offices and factories that went into effect in September, 1896, is about to be rigidly enforced.

The department of health is preparing to carry out the provisions of the new state law factory inspection. The office staff has been kept busy for some time and the work will continue so long as the law is in force. This law obliges all children under 14 and 16 years of age, who are employed at any work, to file certificates furnished by the health department. These certificates contain descriptions of the children, the kind of work at which they are employed, and states whether they are physically able to perform the duties. The department furnishes the certificates after an affidavit has been made by the parents or guardian of the child, stating the place and time of birth. An affidavit must also be made by a school-teacher that the child has attended school for at least one school year. All of those certificates and blank affidavits are provided by the health department. There is a law which provides that no one authorised to administer an oath may make any charge for administering the oaths required by this new law. Children who desire to work only during the school vacation must also obtain certificates which state that fact. No one may legally employ any child who cannot produce the necessary certificate.

The collection of garbage at Chicago, according to the *Medical Recorder*, has been transferred from the department of public works to the department of health. This is a reform upon which the windy city is to be congratulated. Such a revolution in the method of disposal of garbage ought to work to the advantage of any city adopting it, and we hope Buffalo will soon follow Chicago's good example. There can never be too great circumspection exercised in reference to the disposal of garbage. No doubt when garbage receptacles are kept clean and stationed in the midst of sanitary surroundings, a great step in the prevention of disease will be accomplished. Only the well-trained officials of the health department can be made to appreciate this important fact.

Moreover, under the health department we would undoubtedly be relieved of the barbarous practice of collecting garbage between the hours of five and eight A. M. If it were known abroad that such a heathenish custom prevailed in this city, it would no doubt prevent many a man from seeking it as a residence, to say nothing of making us the laughing stock of the rural districts. Even Rochester would be amused that we tolerate such a provincial method. The commissioner of streets should make haste to correct this abuse if he would preserve the good name of our fair city. Unless he does, this may become a question for the health department to interfere with and suppress as it would any other nuisance.

---

Christian science and Schlatterism received a just rebuke in the columns of the *New York Tribune*, November 19, 1896, as follows:

> Doctors are said to be like verbs—regular, irregular and defective; but nobody has yet attempted a scientific classification of the swarm of mystical and supernatural healers to whom the example of Schlatter at the West a few months ago seems to have pointed the way. They rise up in multitudes and pervade all localities near and far, and strangely enough none of them seem to lack followers and believers. It is a curious delusion which reposes faith in their spells, but it is as old as human infirmity, and will probably last as long. The fool we have always with us, and it is not, therefore, a matter of legitimate surprise that we must sometimes have the "healer." He never heals anybody, and individually is soon forgotten, but this type abides and abounds in all lands and seasons, being now and here especially prevalent.

MISS CLARA BARTON, president of the National Red Cross society, who, it will be remembered, went to Armenia last winter to distribute relief among the suffering Christians in that unhappy country, has made public her report. It appears that the Turkish government, which means the Sublime Porte, itself, denied Miss Barton official access to Armenia, but permitted the distribution of relief through her instrumentality as an individual.

(From THE LITERARY DIGEST, New York.)

CLARA BARTON.

The report made by Miss Barton is quite exhaustive and covers the work of the several expeditions sent out from Constantinople. It shows that the work had its inception in the reports of the terrible sufferings endured by the Armenians, gives an account of the sailing of the relief party, of delays in sending out the first expedition on account of the adverse American newspaper comment concerning the Turkish government, and speaks of the various relief expeditions. Miss Barton returns thanks to the press of the United States, to the contributors to the relief fund and to other agencies for aid in carrying out the purposes of the mission.

In conclusion, she says that notwithstanding all that has been done through all agencies, infinitely more remains to be done by someone, for " between the Archipelago and the Caspian seas, the

Black and the Mediterranean,'' she continues, "are today living a million and a half of people of the Armenian race, existing under the ordinance of at least semi-civilisation and professing the religion of Jesus Christ. According to the stated estimates of intelligent and impartial observers of various countries and concurred in by our own agents, whose observations have been unrestricted, from 100,000 to 200,000 of these persons, men, women and children, are destitute of shelter, raiment, fire, food, medicines, the comforts that tend to make human life preservable, or any means of obtaining them, save through the charitable beneficence of the world. The same estimates concur in the statement that without such outside support at least 50,000 of those persons will have died of starvation or perished through accumulated hardships before the 1st of May, 1897.

"None of us has found any better medium for the dispensation of charitable relief than the faithful missionaries already on the ground and our Government officers, whose present course bespeaks their elective interest."

The report of George H. Pullman, financial secretary, shows that there was expended on the relief mission a total of $116,326, of which $7,526 was on account of administration.

We take from the *Literary Digest* the following sketch of Miss Barton's life :

Clara Barton was born in Oxford, Mass., in 1838. She had a thorough education in the public schools of that city, supplemented by a course of study at Clinton, N. Y. For some time she was a teacher in the public schools of Oxford, and subsequently was principal of the first public school at Bordentown, N. J. She was engaged in the Patent Office at Washington in 1861, when the war introduced her to the work that has made her name famous in all lands. Resigning her position in the Patent Office, she devoted herself exclusively to hospital work. As the need increased, she hired a vehicle and went to the scene of the slaughter—Culpepper Court House being her first destination.

Miss Barton was on the battle-fields of Cedar Mountain, Antietam, Fredericksburg, Falmouth, and at the siege of Charleston. How many lives were saved in those scenes of slaughter through her prompt ministrations none can estimate. With her band of trained nurses she did noble service and continued in it to the end of the war.

With a frame exhausted by continuous labor she went, by the advice of her physicians, to Europe to recuperate. She was there when the Franco-Prussian war broke out, and she immediately offered her services. At Metz, in Paris, and in other scenes of the conflict, she ministered to the wounded and comforted the dying. The Emperor of Germany acknowledged her services by the presentation of the Order of the Iron Cross, and other distinguished personages gave her grateful proofs of tangible esteem. Since then, as president of the American Red Cross Society, she has rendered beneficent services in the Ohio floods, the Michigan fires, the Charlestown earthquake, the Johnstown flood and other calamities of national import.

## Figures and Tables

**Figure f1:**